# A Novel *CDC73* Gene Mutation in Hyperparathyroidism Jaw Tumor Syndrome Associated With Ectopic-pelvic Kidney

**DOI:** 10.1210/jcemcr/luad098

**Published:** 2023-08-14

**Authors:** Vijay Sheker Reddy Danda, Vivek Kyatham, Srinivas Rao Paidipally, Sharmila Palli

**Affiliations:** Department of Endocrinology, Gandhi Medical College/Hospital, KNR University of Health Sciences, Hyderabad, Telangana 500003, India; Department of Endocrinology, Gandhi Medical College/Hospital, KNR University of Health Sciences, Hyderabad, Telangana 500003, India; Department of Endocrinology, Gandhi Medical College/Hospital, KNR University of Health Sciences, Hyderabad, Telangana 500003, India; Department of Endocrinology, Gandhi Medical College/Hospital, KNR University of Health Sciences, Hyderabad, Telangana 500003, India

**Keywords:** novel, hyperparathyroidism jaw tumor syndrome, ectopic kidney

## Abstract

A 21-year-old woman presented with polyuria, fragility fractures, and a history of recurrent renal calculi, which was also present in her maternal aunt. Examination revealed an oval palpable mass in the neck. Biochemistry revealed a grossly elevated serum calcium, PTH, and serum alkaline phosphatase with low serum phosphorous, suggestive of primary hyperparathyroidism. Ultrasonography of the neck and parathyroid scintigraphy localized a large lesion arising from the right posterior and inferior aspect of the thyroid gland, suggesting a parathyroid tumor. Parathyroid carcinoma was suspected based on the severe clinical manifestations. A computed tomography scan of the abdomen revealed cysts in the kidneys, bilateral medullary nephrocalcinosis, left ectopic-pelvic kidney, and lytic lesions in the iliac bone. The patient underwent a right inferior parathyroidectomy with normalization of serum calcium postoperatively. Histopathologic examination revealed a parathyroid adenoma, which was contrary to the expectation. Whole exome sequencing in the index case revealed a novel 99-bp heterozygous insertion, likely pathogenic variant in the exon 2 of CDC73 gene causing hyperparathyroidism jaw tumor syndrome. Here, we report a rare case of hyperparathyroidism jaw tumor syndrome that presented with severe hypercalcemia, renal cysts, and an ectopic-pelvic kidney without jaw tumor or uterine abnormalities.

## Introduction

Hyperparathyroidism jaw tumor syndrome (HPT-JT) (OMIM#145001) is a rare autosomal dominant inherited syndrome, with variable penetrance and expressivity with hyperparathyroidism (HPT) being the most penetrant feature. Other clinical features include cemento-ossifying fibromas of the maxilla and mandible, renal lesions, and uterine tumors in women [1]. HPT-JT occurs because of germline inactivating mutations in the tumor suppressor gene *CDC73* (formerly HRPT2). It is composed of 17 exons located on chromosome 1q31.2 encoding a 531-amino acid protein referred to as parafibromin [2]. The majority of germline CDC73 mutations occur within the coding regions, especially in exons 1, 2, and 7, and the remaining may have promoter region abnormalities, whole exon or gene deletions, or epigenetic modifications. More than 75% of the reported CDC73 mutations are frameshift and nonsense mutations, resulting in the truncation of the parafibromin protein or loss of the translated protein through nonsense-mediated mRNA decay [3]. Here, we report a novel CDC73 germline frameshift mutation in a patient with HPT-JT who presented with familial isolated hyperparathyroidism, renal cysts, and an ectopic-pelvic kidney.

## Case Presentation

**Figure 1.**
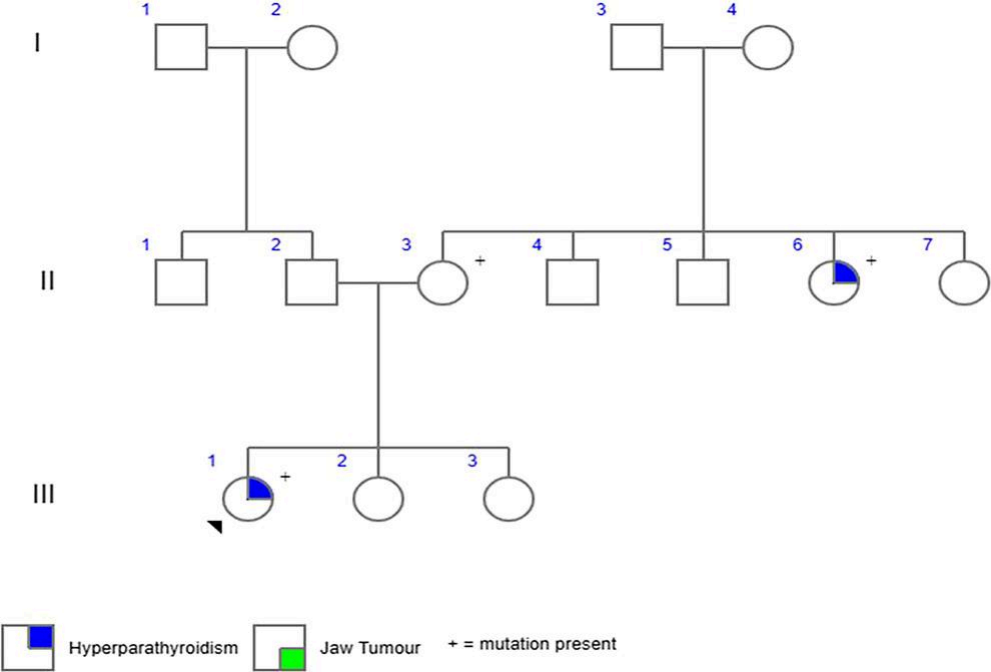
Family tree of the patient.

A 21-year-old female presented with polyuria, polydipsia, constipation, and weight loss of 11 kg in the past year. There was a history of recurrent renal calculi, proximal myopathy, and fragility fractures of the humerus and ulna. She did not give any history of jaw swellings or menstrual irregularities. She was born out of a nonconsanguineous marriage with a history of renal calculi in her maternal aunt and was found to have a neck nodule for which she underwent surgery at 30 years of age (Fig. 1).

## Diagnostic Assessment

**Figure 2.**
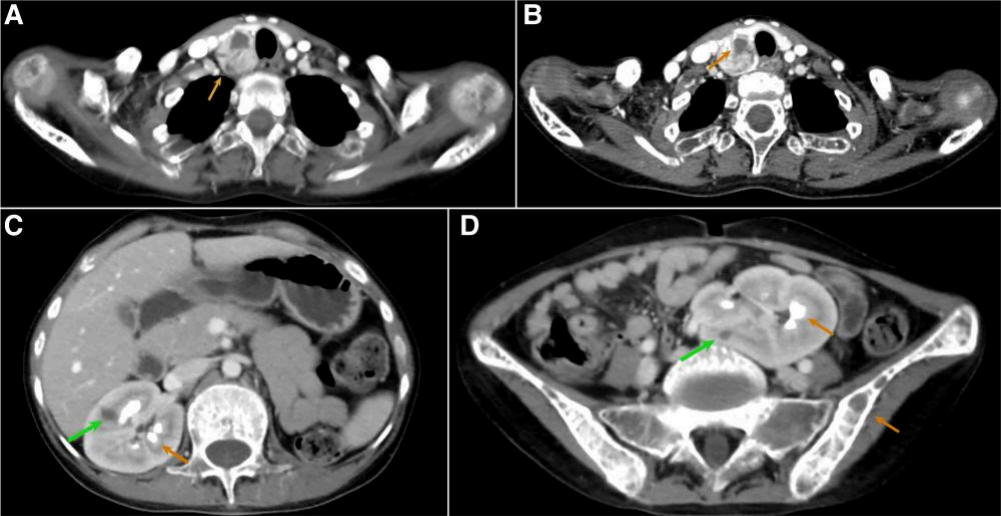
Contrast enhanced CT scan showing (A, B) a large parathyroid lesion arising from the posterior of right thyroid gland, which is intensely enhancing with a cystic component (orange arrow), (C) medullary nephrocalcinosis with a renal cyst (green arrow), (D) ectopic left kidney in pelvis with a renal cyst (green arrow), medullary nephrocalcinosis, and a lytic lesion in iliac bone.

On examination, she was severely underweight with a body mass index of 15.8 kg/m^2^. A 3 cm × 2 cm, oval mass, firm in consistency, was palpable in right lower pole of the thyroid. She had pectus carinatum, scoliosis, and kyphosis along with bony tenderness. Laboratory data at admission revealed severe hypercalcemia (3.77 mmol/L [15.1 mg/dL], normal 2.15-2.54 mmol/L) and highly elevated intact PTH (178.8 pmol/L [1687 pg/mL], normal 1.6-6.9 pmol/L), respectively. Serum alkaline phosphatase was grossly elevated (943 IU/L, normal 40-129 IU/L) with low phosphorous (0.77 mmol/L [2.4 mg/dL], normal range 0.87-1.36 mmol/L) and 25(OH) vitamin D3 (32.95 nmol/L [13.2 ng/mL], normal 74.8-199 nmol/L). Her 24-hour urine calcium level was 3 mmol/L [120 mg] with a calcium/creatinine clearance ratio of 0.012. Ultrasonography of the neck revealed a 3.8 cm × 1.4 cm × 2.2 cm well-defined, oval, hypoechoic, solid lesion with multiple anechoic cystic areas noted within, showing increased internal vascularity, posterior to the inferior pole of right thyroid, which was suggestive of a parathyroid adenoma. This was confirmed by a ^99^Technitium Sestamibi scan that showed increased uptake. Contrast-enhanced computed tomography (CT) of the neck showed a similarly sized, intensely enhancing solid lesion (Fig. 2A) with few nonenhancing cystic areas within (Fig. 2B).

**Figure 3.**
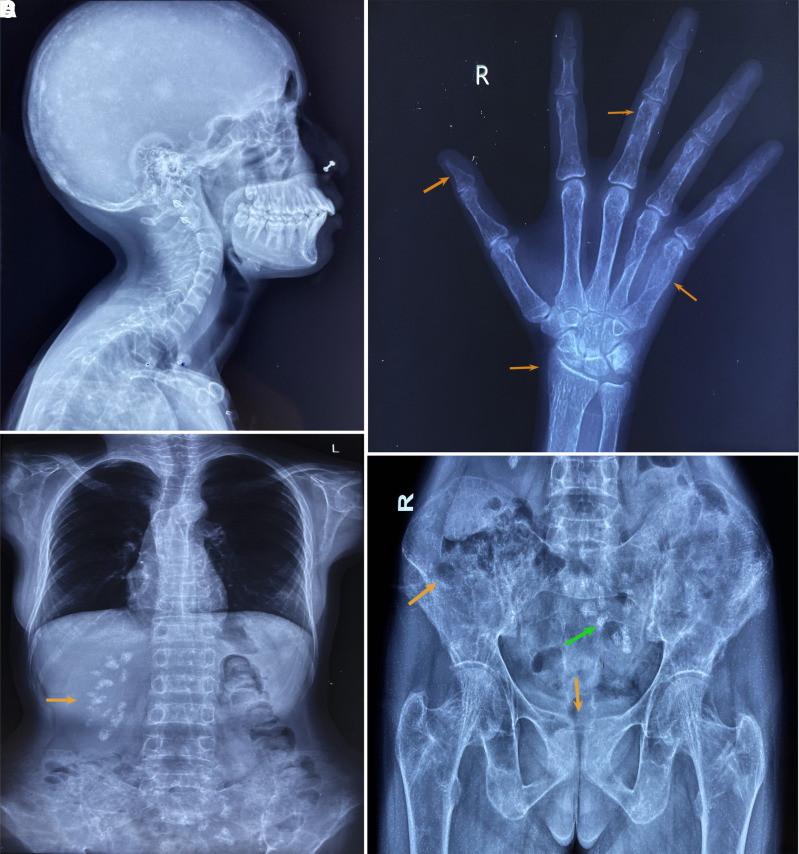
Skeletal radiographs showing (A) salt and pepper skull, (B) acro-osteolysis, sub-periosteal resorption of phalanges and distal radius, and lytic lesion in the 5th metacarpal, (C) medullary nephrocalcinosis of right kidney, (D) ectopic kidney with medullary nephrocalcinosis (green arrow), lytic lesion in iliac bone, and symphysis pubis subchondral erosion (orange arrows).

**Figure 4.**
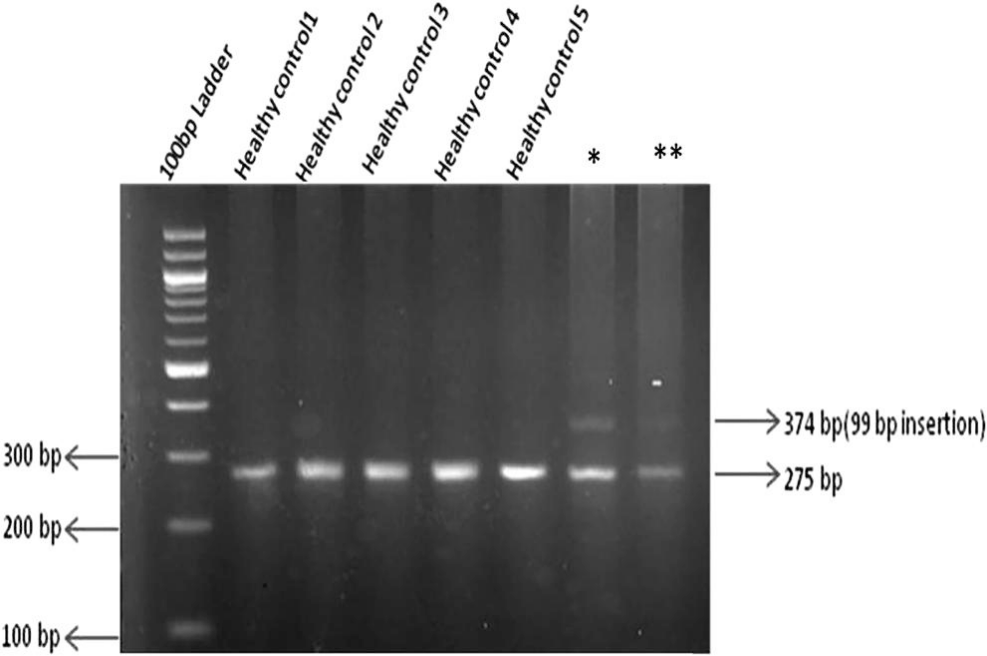
Targeted mutational analysis of CDC73 gene by agarose gel electrophoresis showing a heterozygous insertion (_*_) (1 insertion present only in 1 copy) of 99 bp on chromosome 1.

Dual-energy X-ray absorptiometry bone mineral density (HOLOGIC, Discovery Wi, %*CV* 1%-2%) *Z* score was −2.4 at the lumbar spine, −2.8 at distal radius, and −2.6 at the hip, with a corresponding bone mineral density (g/cm^2^) of 0.762, 0.759, and 0.726, respectively, which is suggestive of osteoporosis. Skeletal radiographs revealed salt and pepper skull (Fig. 3A), acro-osteolysis (Fig. 3B), lytic lesion in the 5th metacarpal (Fig. 3B), humerus, and iliac bone (Fig. 3C), and bilateral medullary nephrocalcinosis with an ectopic left kidney (Fig. 3D) along with diffuse osteopenia. Contrast-enhanced CT scan of the abdomen revealed cysts in both kidneys (Fig. 2C) and bilateral medullary nephrocalcinosis with an ectopic left kidney in the pelvis (Fig. 2D). Imaging did not reveal jaw tumor or uterine abnormalities. Hypercalcemia was managed medically and after adequate replacement of vitamin D3, she underwent right inferior parathyroidectomy followed by normalization of serum calcium levels postoperatively. Histopathologic examination revealed a well-encapsulated nodular mass with cells arranged in solid sheets and a follicular pattern, composed of oxyphilic cells with abundant eosinophilic granular cytoplasm and central round nuclei and nucleoli and other areas composed of chief cells with pale eosinophilic to clear cytoplasm and centrally placed vesicular nucleus. Compressed normal parathyroid tissue was seen just beneath the capsule. There was no evidence of lymphovascular or capsular invasion, thereby giving the impression of a parathyroid adenoma. Whole exome sequencing (WES) was performed in the index case. ACMG guidelines were followed in reporting genetic variants [6]. A heterozygous 99-bp insertion variant (Depth:31X); c.191_192insAATAAAATATTAATAAAATATTTTATACTTAATATTTTATACTTAATATTTTATACTTAATATTTTATACTTAATAATTAAGTATTTTATACTTAATAA, (p.Asn65_Phe531delinsIleLysTyr) in the exon 2 of CDC73 gene (NM_024529.5) was identified (Fig. 4). The variant is not present in publicly available population databases (1000 genomes, EVS, ExAC, gnomAD, dbSNP, and Indian exome database). The variant has not been previously reported to ClinVar, Human Genome Mutation Database, or OMIM databases in affected individuals. In silico pathogenicity prediction programs such as MutationTaster2 and CADD predicted this variant to be likely pathogenic. WES and agarose gel electrophoresis both confirmed that there is a heterozygous insertion of 99 bp in the patient's mother and maternal aunt. However, because of many repetitive sequences, the site was not ideal for Sanger sequencing. Multiple attempts to sequence the insert resulted in a noisy electrophoregram that could not be interpreted.

## Treatment

In the hospital, the patient was treated with IV fluids (normal saline at 100-200 mL/hour for 24 hours) and adequate hydration was ensured before giving injection furosemide (40 mg IV single dose), which was considered in view of severe hypercalcemia. Injection calcitonin (4 IU/kg, subcutaneously twice daily for 2 days) along with injection zolendronic acid (4 mg IV) was given to normalize serum calcium. After correction of serum calcium, 25(OH) vitamin D3 was given at a dose of 60 000 IU per week for 4 weeks and calcitriol was given orally (0.25 µg twice) on the day before the right inferior parathyroidectomy.

## Outcome and Follow-up

PTH decreased by >50% 10 minutes after surgery, and serum calcium normalized postoperatively after 48 hours. Serum calcium and PTH were normal until the last follow-up at 18 months with no recurrences.

## Discussion

Here, we report a rare case of HPT-JT in a young female who presented with a large solitary cystic parathyroid adenoma, with severe skeletal manifestations, medullary nephrocalcinosis, renal cysts, and an ectopic left kidney in the pelvis. WES revealed a novel 99-bp insertion in exon 2, which was classified as a likely pathogenic variant according to ACMG guidelines [4]. This suggests that the presentation of HPT in HPT-JT syndrome may be more severe in comparison to sporadic HPT. In contrast to other forms of hereditary HPT, a single parathyroid gland is usually involved more frequently than are multiple glandular involvements. Histopathology commonly demonstrates a single benign parathyroid adenoma that is often cystic and has atypical features [5]. HPT-JT is associated with a higher prevalence of parathyroid carcinomas (∼21.6%), contrary to other hereditary HPT syndromes [6]. Very high serum calcium (>12 mg/dL) and PTH levels (>3× the upper limit of normal), and parathyroid lesions greater than 3 cm should raise suspicion for parathyroid carcinoma. Given the clinical presentation of severe hypercalcemia and grossly elevated PTH with a palpable neck mass, PTH carcinoma was suspected but the histopathology revealed a benign adenoma. There is a large overlap in clinical and biochemical characteristics of parathyroid adenoma and carcinoma and a majority are diagnosed only through histopathology leading to a diagnostic dilemma. Though no genotype-phenotype correlations have been established, mutations causing gross parafibromin defects are more likely to be associated with the classical HPT-JT phenotype rather than missense mutations that may present as familial isolated HPT [1]. However, contrary to expectations, the patient presented with familial isolated hyperparathyroidism. Testing for the CDC73 mutation is indicated in the presence of familial HPT, HPT with young age onset (<40 years), multiglandular involvement, cystic, atypical, or malignant parathyroid involvement, coexisting ossifying jaw fibroma, renal, or uterine tumors. Selective parathyroidectomy has been advocated if the imaging findings suggest a single-gland involvement without suspicion of parathyroid carcinoma. Despite the name suggesting jaw tumor, it occurs in only 30% to 40% of cases of HPT-JT syndrome. Renal involvement occurs in 15% of cases with HPT-JT syndrome, with cystic kidney disease being the most common followed by hamartomas, Wilms' tumors, and mixed epithelial-stromal tumors [1]. Renal cystic disease is variable and ranges from a few minor cysts to bilateral polycystic disease presenting with end-stage renal disease [7]. Genotype-phenotype correlation has not been reported for Wilms' tumor and renal cysts. However, a specific CDC73 missense mutation, c.3G > A, p.M1I, can present as renal mixed epithelial-stromal tumors and uterine tumors without other manifestations of HPT-JT syndrome [8]. Variable penetrance and expressivity of CDC73 mutation is demonstrated by ∼20% of genetically confirmed *CDC73* mutation-positive subjects lacking any clinical manifestations of HPT-JT [9]. Because the penetrance of clinical manifestations of HPT-JT increases with age, lifelong surveillance of asymptomatic CDC73 mutation carriers is recommended. There are no case reports on associations between ectopic kidneys and HPT-JT syndrome. Isolated ectopic kidney is not associated with increased malignancy risk, though rare case reports of renal cell carcinoma have been described [10]. The effect of the CDC73 mutation on the malignancy risk of ectopic kidneys is not known. Whether ectopic kidney is an incidental finding or a manifestation of the novel mutation is yet to be determined.

## Learning Points

- Hyperparathyroidism jaw tumor syndrome (HPT-JT) syndrome can present with familial isolated hyperparathyroidism even without jaw tumors and uterine abnormalities.
- Young age of onset and large solitary cystic parathyroid adenoma with severe hyperparathyroidism should arouse suspicion for HPT-JT syndrome.
- Cystic kidney disease is a common renal manifestation and ranges from a few minor cysts to bilateral polycystic disease. Ectopic kidney may be a part of spectrum of renal manifestations.
- As the penetrance of clinical manifestations of HPT-JT increases with age lifelong surveillance of asymptomatic CDC73 mutation carriers is recommended.

## Data Availability

Data sharing is not applicable to this article as no data sets were generated or analyzed during the present study.

## Abbreviations

CT: computed tomography
HPT: hyperparathyroidism
HPT-JT: hyperparathyroidism jaw tumor syndrome
WES: whole exome sequencing

## References

[1] Torresan F, Iacobone M. Clinical features, treatment, and surveillance of hyperparathyroidism-jaw tumor syndrome: an up-to-date and review of the literature. Int J Endocrinol. 2019;2019:1761030. 31929790 10.1155/2019/1761030 PMC6935818

[2] Carpten JD, Robbins CM, Villablanca A, et al HRPT2, encoding parafibromin, is mutated in hyperparathyroidism-jaw tumor syndrome. Nat Genet. 2002;32(4):676‐680. 12434154 10.1038/ng1048

[3] Newey PJ, Bowl MR, Cranston T, Thakker RV. Cell division cycle protein 73 homolog (CDC73) mutations in the hyperparathyroidism-jaw tumor syndrome (HPT-JT) and parathyroid tumors. Hum Mutat. 2010;31(3):295‐307. 20052758 10.1002/humu.21188

[4] Richards S, Aziz N, Bale S, et al Standards and guidelines for the interpretation of sequence variants: a joint consensus recommendation of the American College of Medical Genetics and Genomics and the Association for Molecular Pathology. Genet Med. 2015;17(5):405‐424. 25741868 10.1038/gim.2015.30 PMC4544753

[5] Kelly TG, Shattuck TM, Reyes-Mugica M, et al Surveillance for early detection of aggressive parathyroid disease: carcinoma and atypical adenoma in familial isolated hyperparathyroidism associated with a germline HRPT2 mutation. J Bone Miner Res. 2006;21(10):1666‐1671. 16995822 10.1359/jbmr.060702

[6] Iacobone M, Carnaille B, Palazzo FF, Vriens M. Hereditary hyperparathyroidism—a consensus report of the European Society of Endocrine Surgeons (ESES). Langenbecks Arch Surg. 2015;400(8):867‐886. 26450137 10.1007/s00423-015-1342-7

[7] Tan MH, Teh BT. Renal neoplasia in the hyperparathyroidism-jaw tumor syndrome. CurrMol Med. 2004;4(8):895‐897. 10.2174/1566524043359719 15579037

[8] Vocke CD, Ricketts CJ, Ball MW, et al CDC73 germline mutation in a family with mixed epithelial and stromal tumors. Urology. 2019;124:91‐97. 30452964 10.1016/j.urology.2018.11.013 PMC6382532

[9] Li Y, Zhang J, Adikaram PR, et al Genotype of CDC73 germline mutation determines risk of parathyroid cancer. EndocrRelat Cancer. 2020;27(9):483‐494. 10.1530/ERC-20-0149 PMC8802173 32590342

[10] Alokour RK, Ghawanmeh HM, Al-Ghazo M, Lafi TY. Renal cell carcinoma in ectopic-pelvic kidney: a rare case with review of literature. Turk J Urol. 2018;44(5):433‐436. 30487047 10.5152/tud.2018.22058 PMC6134975

